# Percutaneous left atrial appendage closure reduces cost of care independent of the institutional cumulative caseload in patients with non-valvular atrial fibrillation

**DOI:** 10.1007/s12471-022-01675-x

**Published:** 2022-03-29

**Authors:** G. D’Ancona, F. Arslan, E. Safak, D. Weber, H. Ince

**Affiliations:** 1 Department of Cardiology, Vivantes Klinikum Am Urban, Berlin, Germany; 2 Department of Cardiology, Leiden University Medical Centre, Leiden, The Netherlands; 3 Department of Cardiology, Vivantes Klinikum Wenckebach, Berlin, Germany; 4 Department of Cardiology, Rostock University, Rostock, Germany

**Keywords:** Atrial fibrillation, Left atrial appendage, Anticoagulation, Closure, Costs

## Abstract

**Background:**

Data on the impact of the cumulative percutaneous left atrial appendage closure (LAAC) caseload on cardiovascular outpatient and hospitalisation costs are limited.

**Methods:**

The present single-institution analysis includes patients treated consecutively from the beginning of our LAAC experience in January 2012 until December 2016. Pre- and post-LAAC costs for hospitalisation and ambulatory visits were included.

**Results:**

A total of 676 patients underwent percutaneous LAAC (using the Watchman device): 49 (2012), 78 (2013), 211 (2014), 210 (2015), and 129 (2016). LAAC procedural costs were stable over the years (overall median €9639; 2012: €9630; 2013: €10,003; 2014: €9841; 2015: €9394; 2016: €9530; *p* = 0.8) and there was no correlation between cumulative caseload and procedural costs (*p* = 0.9). Although annualised cardiovascular management costs after LAAC were lower than before LAAC (median difference between pre-LAAC and post-LAAC yearly costs: €727; 2012: €235; 2013: €1187; 2014: €716; 2015: €527; 2016: €1052; *p* = 0.5 among years analysed) from the beginning of the cumulative procedural experience, a significant reduction in costs was observed only from 2014 onwards. Institutional cumulative LAAC caseload and year of procedure were not related to the amount of reduction in the costs for cardiovascular care.

**Conclusion:**

LAAC led to cost-of-care savings from the beginning of our institutional procedural experience.

## What’s new?

- We are the first group to investigate actual real-world management costs of percutaneous left atrial appendage closure (LAAC) in patients with non-valvular atrial fibrillation and to focus on the impact of the learning curve.
- Management costs are not learning curve related.
- Cost savings (difference between pre- and post-procedural management costs) have been significant and evident since our very early clinical experience with the procedure.
- Moreover, we are the first group to show an independent relation (inverse) between DRG clinical complexity level and cost-of-care savings after the procedure.
- Finally, we have shown that survival after LAAC is not affected by the caseload or procedural experience.

## Introduction

Atrial fibrillation (AF) is the most common sustained cardiac arrhythmia with a risk of cerebral thromboembolism that rises with the increase in patients’ age and cardiovascular comorbidity [1, 2].

Because the left atrial appendage (LAA) is the most common and clinically relevant source of thrombi in patients with non-valvular AF, its percutaneous closure (LAAC) represents a valid armamentarium in patients with an absolute contraindication to anticoagulation.

Percutaneous LAAC is still not widely implemented in daily clinical practice and is considered a novel procedure. Therefore, outcomes and costs could be impacted by the cumulative LAAC caseload and by the learning curve. In the current analysis we have investigated the impact of the cumulative LAAC caseload on costs, cost-of-care savings, and outcomes. In particular we report on the pre-, intra-, and post-procedural management costs in a consecutive series of patients with thromboembolic non-valvular AF treated with percutaneous LAAC using the Watchman device (Boston Scientific, Marlborough, MA, USA).

## Methods

The Vivantes network of affiliated hospitals in Berlin is the largest community-based hospital chain in Germany, serving an area that includes about one-third of the circa 3.6 million inhabitants of Berlin.

Percutaneous LAAC has been performed within the Vivantes network since January 2012, using the Watchman device. The present analysis includes only consecutive patients treated with LAAC between January 2012 and December 2016. Hospital admissions (including ambulatory visits) to the Vivantes clinics occurring before and after the admission for percutaneous LAAC were identified. Only hospital admissions and ambulatory visits for cardiovascular reasons, including anticoagulation and bleeding management, were selected, and costs were retrieved. The observation period spanned from the first admission with a diagnosis of AF until the last admission, for follow-up post-LAAC or management of cardiovascular complications, or other issues related to LAAC procedure/management.

Demographic, clinical, and treatment cost information regarding the admitted patients was prospectively recorded in a centralised electronic database and was retrospectively analysed.

HAS-BLED (Hypertension, Abnormal kidney and liver function, Stroke, Bleeding, Labile international normalised ratios, Elderly ≥ 65 years, Drugs (like aspirin) and alcohol (more than eight drinks a week)) and CHA_2_DS_2_-VASc scores (Congestive heart failure, Hypertension, Age ≥ 75 years, Diabetes mellitus, Stroke, Vascular disease, Age 65–74 years, Sex category (i.e. female sex)) were computed.

Hospital service fees were calculated and remunerated via the DRG (diagnosis-related groups) system. Details of the DRG’s remuneration system are regulated by the Hospital Financing Act, the Hospital Remuneration Act, and the flat rate case agreement of the self-government partners.

For every patient, the primary diagnosis and secondary diagnoses were recorded and used to compute cumulative and yearly costs for hospital admissions and visits before LAAC, the LAAC procedure itself, and after LAAC. The clinical complexity level (CCL, also known as comorbidity and complication level or severity level) was used, together with the primary diagnosis, to account for the severity of the comorbidities and complications in the secondary diagnoses and for billing to the DRG system. The CCL weighting is divided into four levels according to the comorbidity and morbidity profile (CCL 0 = no complication or comorbidity; CCL 1 = mild complication or comorbidity; CCL 2 = moderately severe complication or comorbidity; CCL3 = severe complication or comorbidity; CCL 4 = very severe complication or comorbidity).

The ethics committee of the University of Applied Sciences of Neubrandenburg (Neubrandenburg, Germany) approved the study under the registration number HSNB/KHM/146/19.

### Statistical analysis

Patients were classified according to the year of the LAAC procedure year (categorical variable) and a consecutive case number was also assigned starting from the first procedure (continuous variable).

Overall costs, procedural costs, pre- and post-LAAC total and yearly (annualised) costs were calculated. Differences between pre- and post-LAAC costs were also computed to identify the possible cost-of-care savings after LAAC.

Analysis of variance (ANOVA) (post hoc Tukey test), non-parametric tests, chi-square and Fisher exact tests were used when appropriate and to compare differences among year of LAAC procedure groups. Multivariate analysis by means of backward linear regression were used to identify independent determinants of cost-of-care savings after LAAC.

Kaplan-Meier survival curves were generated to calculate estimated survival at follow-up and differences in survival according to the year of the LAAC procedure (log rank test).

Data are presented as absolute numbers, percentages, mean ± standard deviation, or median with 75% interquartile range (IQR) as appropriate. Statistical analysis was performed using IBM SPSS version 25 (IBM Corp., Armonk, NY, USA) and the level of significance was set at *p* < 0.05 (two-tailed).

## Results

**Fig. 1:**
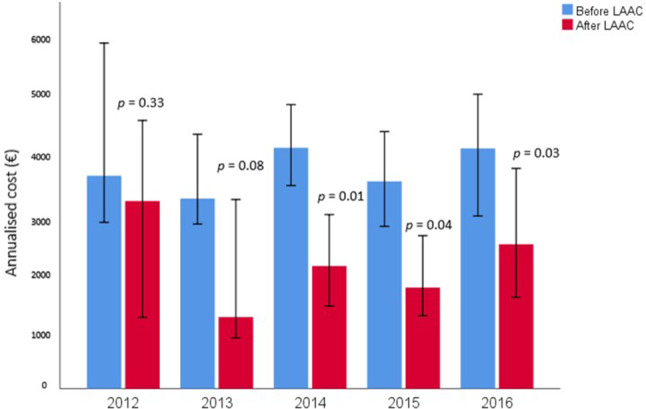
Annualised management costs pre- and post-left atrial appendage closure (*LAAC*) according to implantation year

A total of 676 patients underwent percutaneous LAAC: 49 (2012), 78 (2013), 211 (2014), 210 (2015), and 129 (2016). Tab. 1 summarises the baseline characteristics and in-hospital mortality rate categorised by year of LAAC. No significant differences among groups were reported. In particular, in-hospital outcomes and procedural costs were comparable from the early phase of our experience with LAAC until the most recent procedures. The annualised cost pre-LAAC was 3773 Euros (IQR: 1644–8493) and post-LAAC 2001 Euros (IQR: 260–6913) (*p* < 0.0001). Fig. 1 and Table 2 summarize the annualised costs before and after the LAAC admission according to the year of implantation. The overall median difference between pre- and post-LAAC costs (annualised cost-of-care saving) was €727 (IQR: −2148 to 4229). There was a constant reduction in management costs after LAAC compared to costs pre-LAAC from the beginning of our experience with a constant cost-of-care saving (2012: €235; 2013: €1187; 2014: €716; 2015: €527; 2016: €1052; *p* = 0.5). A significant reduction of management costs was observed only from 2014 onwards (Fig. 1).

**Table 1:** Baseline characteristics, in-hospital mortality rates and actual implantation costs. Data are derived from patient DRG records. *LAAC* left atrial appendage closure

Year of LAAC	Age (years)	Gender (F/M) %	CHA_2_DS_2_-VASc score	HAS-BLED score	In-hospital mortality (%)	Implantation costs (median and IQR) €
2012 (49)	76.0 ± 6.4	46.9/53.1	4.9 ± 1.3	3.3 ± 1.4	0	9630 (9501–9966)
2013 (78)	76.1 ± 7.4	44.9/55.1	4.0 ± 1.4	3.2 ± 1.0	2.6	10,003 (9831–10,106)
2014 (211)	76.0 ± 7.8	40.8/59.2	4.5 ± 1.6	3.6 ± 1.1	1.4	9841 (9613–10,563)
2015 (210)	75.4 ± 8.0	41.4/58.6	4.2 ± 1.5	3.4 ± 1.0	0.5	9394 (9394–9704)
2016 (129)	76.2 ± 7.9	41.1/58.9	4.0 ± 2.6	3.3 ± 1.3	2.3	9530 (9230–9530)
*p*-value	0.8	0.9	0.1	0.08	0.4	0.8

**Table 2:** Annualised management costs pre- and post-left atrial appendage closure (LAAC) according to implantation year

Year LAAC	Annualised costs Pre-LAAC €	Annualised costs Post-LAAC €
2012 (49)	3670 (2366–8567)	3235 (421–8739)
2013 (78)	3276 (2037–7013)	1235 (275–6041)
2014 (211)	4125 (1560–10,395)	2114 (249–6925)
2015 (210)	3572 (1437–8596)	1742 (149–7252)
2016 (129)	4139 (1800–7687)	2489 (265–7115)

**Fig. 2:**
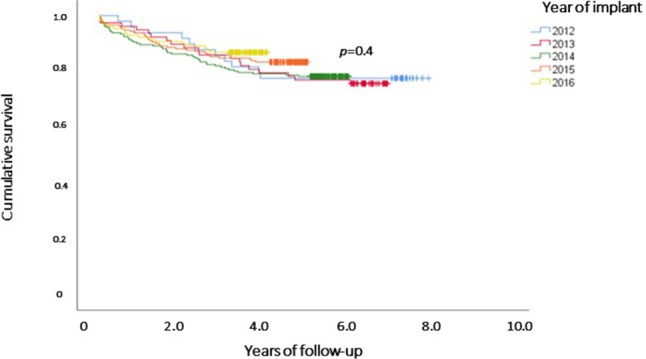
Kaplan-Meier survival curves after left atrial appendage closure and according to implantation year

The median follow-up duration after LAAC was 4.8 years (IQR: 3.6–5.6). Observed all-cause follow-up mortality was 16.9%. Kaplan-Meier analysis revealed no differences in survival between groups stratified by year of implantation (Fig. 2; *p* = 0.4).

**Fig. 3:**
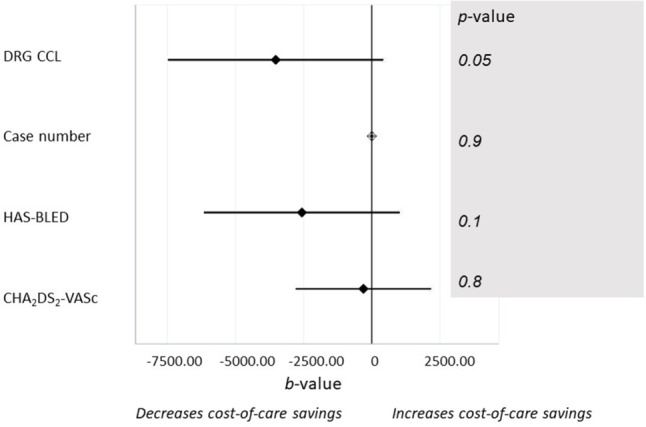
Linear regression: independent determinants of cost-of-care savings after left atrial appendage closure. *DRG* diagnosis-related groups system, *CCL* clinical complexity level

Linear regression analysis revealed that only CCL was an independent determinant of the amount of cost-of-care savings after LAAC (inverse relationship) (Fig. 3).

## Discussion

From our actual and real costs analysis, which considers the development of our institutional experience, it emerges that:

1. Costs are not increased in the initial phase of the learning curve with LAAC. On the contrary, cost-of-care savings are evident from the very early phase of our clinical experience with percutaneous LAAC.
2. There is an inverse and independent relation between CCL, computed using the DRG indications, and cost-of-care savings after LAAC.
3. Survival after LAAC is not affected by caseload/procedural experience.

To the best of our knowledge, we are the first group to investigate actual real-world management costs of percutaneous LAAC and focus on the potential impact of the procedural learning curve. In the current scientific literature, economic evaluations of percutaneous LAAC are derived mainly from mathematical simulations and very seldom from documented costs originating from analysis of DRG documents for specific timeframes [3–5]. In this context, government policymakers and health insurers still consider percutaneous LAAC to be costly, also in light of the fact that scientific evidence supporting LAAC-improved clinical outcomes is still limited. An adequate cost analysis that includes very early phases of the learning curve could increase acceptance regarding the sustainability of this procedure.

The analysis presented herein is the first to demonstrate that outpatient and clinical care costs related to non-valvular AF are not affected by the institutional learning curve with percutaneous LAAC. The reported costs are actual amounts claimed from the German health insurance funds. These costs were derived from the DRG system and hospital claims records and are not based on mathematical modelling or any other assumptive calculations.

Since the introduction of percutaneous LAAC in our hospital network in 2012, procedural costs have been stable, and medical management costs have significantly decreased after treatment, justifying the procedure costs in selected patients with a contraindication to oral anticoagulation. We have previously confirmed that patients undergoing LAAC will cause lower AF-related hospital costs secondary to admissions and outpatient treatments to manage anticoagulation, its iatrogenic effects, and to diagnose and treat cerebrovascular embolism [6]. In fact, an indication for LAAC was strictly and institutionally based upon guidelines [7], and all patients had previously experienced at least one cerebral thromboembolic event, one major bleeding episode, and had an absolute contraindication to prolonged oral anticoagulation. Immediately after LAAC, costs are mainly related to procedural follow-up. With time, and in patients with an extended lifespan, costs are reduced and the chance to achieve cost parity between pre- and post-LAAC costs is increased [6].

As documented in our study, cost-of-care savings are related to the clinical complexity of the single patient, represented in the DRG CCL value, rather than to the institutional cumulative caseload.

The volume-outcome relationship has been proposed for complex surgical procedures and percutaneous treatment of structural heart disease. In particular, a difference in mortality and hospitalisation costs, based on the annual procedural volume, has emerged within groups of hospitals performing transcatheter aortic valve replacement [8]. In the field of percutaneous LAAC, the volume-outcome relationship remains controversial. Badheka et al. showed that in a cohort of 268 LAACs, performed with different closure devices, higher annual hospital volume is associated with safer procedures and consequent lower length of stay and hospitalisation costs [9]. In a more recent and more extensive analysis, including 425 patients undergoing LAAC with the Watchman device, Sawant et al. demonstrated that operator experience does not affect major adverse events and technical success even after adjusting for comorbidities. However, the authors did not present a cost analysis based on case volume and year of the procedure [10]. The use of different closure devices may cause differences in device-related procedural success and costs, which hampers analysis of cost savings.

Finally, we are aware that in the existing literature, management costs for LAAC patients have been compared with costs in patients managed solely with oral anticoagulation. We believe that such a comparison risks including patients with very different risk profiles and conceals the impact of the treatment strategy. In light of this, we have preferred to use every patient as her/his own control and to evaluate the variation in management costs after percutaneous LAAC. Such an analysis was possible only because our network of affiliated clinics serves the large majority of the urban population of Berlin, and, for this reason, we have a very granular picture of treatments performed per single patient over the years.

## Conclusion

In the present work we have shown that since the beginning of our experience with LAAC in 2012, the LAAC implantation costs have remained stable and there has been a constant post-procedural cost-of-care saving. Management costs and cost-of-care savings are independent of the institutional cumulative caseload. These findings are supported by the fact that peri-procedural complications and mortality have remained limited, even at the beginning of our learning curve, and follow-up outcomes have confirmed the safety of this innovative technology.
